# Rodenticide poisoning leading to cerebral hemorrhage: A case report

**DOI:** 10.1097/MD.0000000000036971

**Published:** 2024-02-16

**Authors:** Qian-kun He, Yuan-hua Wu, Xiao-ying Lu, Ming-wei Liu

**Affiliations:** a Department of Neurology, The First Affiliated Hospital of Guizhou University of Traditional Chinese Medicine, Guiyang, Guizhou, China; b Department of Neurology, The First Affiliated Hospital of Kunming Medical University, Kunming, Yunnan, China; c Department of Emergency Medicine, The First Affiliated Hospital of Kunming Medical University, Kunming, Yunnan, China; d Department of Emergency Medicine, The First Affiliated Hospital of Guizhou University of Traditional Chinese Medicine, Guiyang, Guizhou, China.

**Keywords:** case report, cerebral hemorrhage, diagnosis, rodenticide poisoning, treatment

## Abstract

**Rationale:**

Anticoagulant rodenticides (ARs) are a substantial fraction of murine types. AR poisoning causes bleeding from the skin, mucous membranes, and multiple organs. However, reports of AR-induced cerebral hemorrhage are scarce.

**Patient concerns:**

A 40-year-old male presented with dizziness, headache, and limb weakness for 5 days and with coagulopathy. Two days prior to the onset of these symptoms, the patient was exposed to dead mice.

**Diagnoses:**

Rodenticide intoxication-induced cerebral hemorrhage.

**Interventions:**

Vitamin K1 infusion, administration of dehydrating agents to reduce intracranial pressure, and correction of acid-base and electrolyte imbalances.

**Outcomes:**

After 9 days of treatment, the patient’s symptoms were relieved, and reexamination revealed that coagulation parameters returned to normal levels. The patient was eventually discharged for observation with oral vitamin K1.

**Conclusions:**

Rodenticide poisoning can lead to intracerebral hemorrhage, and treatment with vitamin K1 infusion is effective.

**Lesson:**

Rodenticide poisoning-induced cerebral hemorrhage is rarely reported. Because its symptoms are nonspecific, it is easy to miss the diagnosis or misdiagnose. When patients present with direct and indirect symptoms such as dizziness, headache, and limb weakness, rodenticide poisoning should be considered. Coagulation function and head computed tomography or magnetic resonance imaging examination should be performed at the earliest to confirm the diagnosis and provide timely treatment.

## 1. Introduction

Plague of rats has always been a challenging problem for humans, and rodenticides are highly toxic, with no specific detoxifying drugs after the poisoning, and have, therefore, been restricted from use.^[1]^ Anticoagulant rodenticides (ARs), particularly second-generation rodenticides such as bromadiolone and brodifacoum, which destroy the coagulation mechanism in rats, are currently the most widely used rodenticides and are also one of the varieties reportedly causing rodenticide poisoning in clinical practice.^[2]^ Rodenticide poisoning in humans can occur through various routes such as accidental ingestion and intentional ingestion and clinically manifests as acquired coagulopathy. According to the American Center for Toxicity Surveillance, as many as 16,000 cases of rodenticide poisoning occurred in the United States in 2008 alone.^[3]^ Although the overall incidence has comparatively decreased in recent years, poisoning from rodenticides such as brodifacoum has increased^.[4]^

According to the development of ARs, they are divided into first-generation (such as warfarin and diphacinone) and second-generation (such as bromadiolone and dalong) ARs. While first-generation ARs have been gradually withdrawn from the market, second-generation ARs continue to be marketed as rodenticides since 1970 and are widely used for rodenticide control in agriculture, food establishments such as catering, and other industries.^[5]^

AR poisoning tends to have a more insidious onset with manifestations such as unexplained skin, mucous membrane, or organ bleeding; bleeding from >1 site, such as gingival bleeding, epistaxis, skin ecchymosis, hematuria, melena, hematemesis, and menorrhagia; severe shock; and coma. King et al^[6]^ analyzed the clinical manifestations of 174 patients with long-acting AR (LAAR) poisoning and found that hematuria (59.34%), gingival bleeding (52.30%), epistaxis (41.24%), gastrointestinal bleeding (40.23%), and spontaneous ecchymosis (38.22%) occurred at a higher rate than intracerebral hemorrhage. In patients with AR poisoning, the symptoms are not typical; therefore, diagnosis is easily missed or misdiagnosed. Here, we report the diagnosis and treatment of a patient with cerebral hemorrhage caused by rodenticide poisoning. We believe that our findings will help guide clinicians to accurately diagnose and provide timely treatment to patients, thereby reducing missed diagnoses or misdiagnoses and enable such patients to receive timely and effective treatment.

## 2. Case report

### 2.1. Ethics approval and consent to participate

Informed written consent was obtained from the patient and their parents for the publication of this case report and the accompanying images.

This study was reviewed and approved by the local ethics committee of First Affiliated Hospital of Kunming Medical University. Procedures followed were in accordance with the Helsinki Declaration of 1975, as revised in 2000.

### 2.2. Medical history

A 40-year-old male patient presented with dizziness, headache, and weakness of both lower limbs without obvious inducement for 4 days and without coma, convulsion, or incontinence. A head computed tomography (CT) examination performed at a local hospital showed intracranial hemorrhage. Coagulation examination showed prothrombin time (PT) >120 seconds and activated partial thromboplastin time (APTT) 152.6 seconds. Due to abnormal coagulation function combined with severe cerebral hemorrhage, the risk of death is high, the patient was urgently transferred to the First Affiliated Hospital of Kunming Medical University for further treatment.

### 2.3. Past medical history

Two days prior to the onset of the symptoms, the patient was exposed to dead mice in the field. He reported no prior history of diabetes; cardiovascular, cerebrovascular, lung, kidney, endocrine system, and other important organ diseases; infectious diseases; trauma; surgery; blood transfusion; or allergy. Vaccination was unknown.

### 2.4. Physical examination

The patient’s general condition was good, with the following vital sign recordings: temperature 36.4°C, pulse 70 bpm, respiratory rate 18 times/min, heart rate 93 bpm (with regular rhythm), and blood pressure 127/79 mm Hg. There was no systemic superficial lymph node or thyroid gland enlargement. No head or facial deformity was observed, with normal bilateral eyeballs. Furthermore, there were no thoracic deformities, coarse breath sounds in the bilateral lung, dry or moist rales heard, expansion of the heart border, or pathological murmur heard in any of the valve areas. The abdomen was flat and soft without tenderness or rebound tenderness, liver static was not palpable, and bowel sounds were normal. Special examination showed clear consciousness, poor mental state (Glasgow Coma Scale score 15 points), fluent speech, no aphasia apraxia, no dysarthria, and cooperative physical examination. Orientation, calculation, and memory phase measurements were normal. There was no stiffness of the neck and no resistance; however, the Kernig and Brudzinski signs were negative. Bilateral pupils were equal and round (d = 3 mm), direct and indirect light reflexes were present, bilateral movements were fair, diplopia nystagmus was absent, and coarse visual field visual acuity measurements were fair. Bilateral frontal lines were symmetrical, bilateral nasolabial folds were symmetrical when eyes were firmly closed, no deviation at the angle of the mouth, the tongue was centered, the bilateral soft palate was lifted, the uvula was centered, bilateral pharyngeal reflexes (−), and head up shoulder and neck rotation were strong. Furthermore, the following were observed: no atrophy of limb and trunk muscles; grade V distal proximal muscle strength of bilateral upper limb; grade IV proximal distal muscle strength of bilateral lower limb; tendon reflexes (−); negative pathological signs; rough measurement of pain and temperature tactile sensation; position sensation and vibration sensation of limbs; normal nutrition of skin, hair, and fingernails; normal sweating; normal fecal and urinary function; no abnormal skin scratches; modified Rankin Scale score 4; and no obvious anxiety and depression.

### 2.5. Laboratory data

On July 12, 2020, blood routine showed the following: white blood cells 9.07 × 10^9^/L, neutrophils 70.4%, lymphocytes 20.3%, red blood cells 4.92 × 10^12^/L, hemoglobin 149 g/L, platelets 203 × 10^9^/L, procalcitonin < 0.05 mg/L, and C-reactive protein 26.5 pg/L. Blood coagulation parameters were as follows: PT 100.0 seconds, international normalized ratio 13.33, PT ratio 0.91, reagent sensitivity index 1.26, fibrinogen 4.37 g/L, coagulation time 16.7 seconds, and APTT 157.6 seconds. The fibrinolysis system test showed fibrin degradation products 1.8 mg/L, D-dimer 0.45 mg/L, and antithrombin III 84%. The concentration of bromadiolone, a rodenticide, was detected to be 3.478 μg/mL in the patient’s blood for testing. In addition, coagulation liver and kidney function, electrolyte, myocardial enzyme, and urine analyses showed no abnormalities. Tests for systemic lupus erythematosus, rheumatoid-associated antibodies, anticardiolipin antibodies, and antineutrophil cytoplasmic antibodies were negative. Novel coronavirus nucleic acid also tested negative.

### 2.6. Imaging data

**Figure 1:**
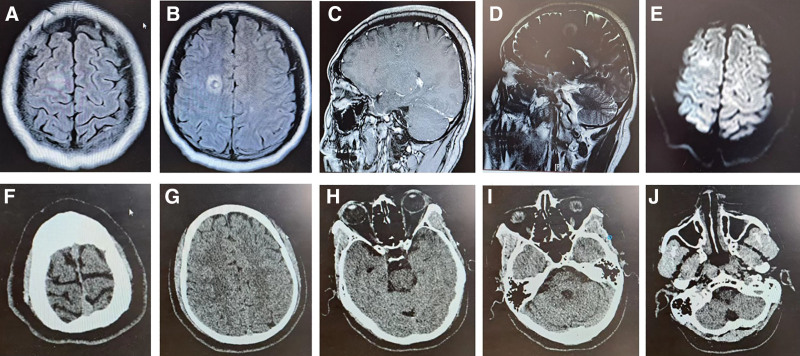
Changes in imaging of head bleeding lesions in patients before and after treatment. (A–E) Changes in MRI at patient admission. (H–J) Head CT changes during follow-up 6 mo after discharge. CT = computed tomography, MRI = magnetic resonance imaging.

A cranial magnetic resonance imaging (MRI) scan performed on July 13, 2020, showed a right centrum semiovale lesion with intralesional hemorrhage (Figure 1A–E). In addition, 2 small strips of abnormal signals were observed around this lesion, and the possibility of hemorrhagic changes was also considered (Figure 1A–E). Symmetrical abnormal signals were observed in the corpus callosum, posterior limbs of the bilateral internal capsule, bilateral centrum semiovale, and paraventricular region on diffusion weighted imaging, suggesting extensive white matter lesions (Figure 1A–E). There was no intracranial arteriovenous malformations.

### 2.7. Diagnosis and treatment

According to the patient’s medical history, signs and symptoms, and laboratory data, he was diagnosed with rodenticide poisoning, coagulation disorder, and cerebral hemorrhage. After admission, the patient was given vitamin K1 40 mg once daily, intravenous drip, dehydrating agents to reduce intracranial pressure, and correction of acid-base and electrolyte imbalances. The changes in coagulation parameters (Table 1) and factors (Table 2) were dynamically observed. Head CT and MRI changes were reexamined at any time if the patient had conscious changes. After 14 days of treatment, dizziness and headache were significantly alleviated, and no abnormalities in coagulation parameters or factors were observed. The patient was discharged home with oral vitamin K1 for 6 months.

**Table 1:** Changes in coagulation indicators before and after treatment.

Coagulation indicators	July 12, 2020	July 13, 2020	July 14, 2020	July 15, 2020	July 17, 2020	July 24, 2020	January 14, 2021
PT(s)	100	22	18.9	15.1	14.3	15.7	14.7
ISI	1.26	1.26	1.26	1.26	1.26	1.26	1.26
INR	13.3	1.98	1.64	1.23	1.15	1.30	1.21
PTR (%)	9.38	1.72	1.48	1.18	1.12	1.26	1.16
FIB (g/L)	4.37	5.15	4.45	4.29	3.49	2.91	4.34
TT (s)	16.7	15.9	16.4	15	15.3	17.5	14.7
APTT (s)	157.6	60	44.3	40	42	38.3	37.5

APTT = activated partial thromboplastin time, FIB = fibrinogen, INR = international normalized ratio, ISI = reagent sensitivity index, PT = prothrombin time, PTR = prothrombin time ratio, TT = coagulation time.

**Table 2:** Changes in the activity of coagulation factors in plasma before and after treatment.

Coagulation factors	July 12, 2020	July 14, 2020	July 17, 2020	July 23, 2020	January 14, 2021
V (%)	104	113	117	99	112
VII (%)	30	73	86	70	89
VIII (%)	200	198	212	183	207
IX (%)	26	61	60	58	64

### 2.8. Follow-up after treatment

A follow-up at 6 months after discharge found that the patient experienced no discomfort. Head CT showed that intracranial hematoma was absorbed without abnormality (Fig. 1F,G). As a result, vitamin K1 was stopped for 1 week, and coagulation parameters and factors showed no abnormality (Tables 1 and 2). Then, vitamin K1 was stopped for 1 month, and there remained no coagulation abnormality. Based on these findings, vitamin K1 was stopped completely. During the treatment period, advanced and unanticipated events were not detected.

## 3. Discussion

Since 2004, the sale of tetramethylene disulfone tetramine and fluoroacetamide in China has been put in force. Nonetheless, the number of poisoning cases from other types of rodenticides has gradually increased, of which ARs are the most common. A 10-year epidemiological survey of rodenticide poisoning in Hainan General Hospital showed that the proportion of AR poisoning was 57.5%, and the misdiagnosis rate was 15.4%.^[7]^ From 2008 to 2012, there were nearly 10,000 cases/year of LAAR poisoning in the United States, mostly 0.005% bromadiolone and brodifacoum.^[8]^ As AR use increased, accidental exposure to poison has also increased. Epidemiological data from 1987 to 2012 by the American Association of Poison Control Centers indicated that poisoned patients are exposed an average of 10,413 times/year, and the proportion of accidental exposures was 95.6%.^[8]^ Because LAAR poisoning has an insidious onset and often presents with mucocutaneous bleeding and vitamin K1-dependent coagulopathy, it is often misdiagnosed and mistreated. Epidemiological studies have shown that the incidence of human rodenticide poisoning has been increasing worldwide in the past decade, with the most common cause being accidental ingestion, especially in children, and other causes include suicide, mental illness, Monjosen syndrome, and murder.^[9]^ In the present study, the patient presented with dizziness, headache, and limb weakness and had no history of direct exposure to rat poison or suicidality. However, the patient had a history of picking up “dead mice” in the field 2 days before the onset of the disease. Therefore, rodenticide poisoning-induced cerebral hemorrhage was not considered, which resulted in misdiagnosis and mistreatment, and vitamin K1 treatment was not administrated on time.

Some of the clinical manifestations of AR poisoning include systemic mucosal and organ bleeding, common hematuria, skin ecchymosis, gingival bleeding, gastrointestinal bleeding, epistaxis, and hemoptysis.^[10,11]^ ARs inhibit the production of vitamin K by inhibiting the activity of vitamin K 2, 3-epoxide reductase. In addition, the glutamic acid residues of vitamin K-dependent coagulins cannot be carboxylated, thus affecting the activation of coagulation factor II, VII, IX, and X, making them unable to exert their coagulant activity.^[12]^ AR metabolite benzylidene acetone may also directly damage the capillary wall. As a result, the fragility and permeability of the vascular wall are enhanced, resulting in more bleeding. Reportedly, bleeding resulting from the anticoagulant effects of rodenticides can last from 51 days to 13 months.^[13]^ ARs have a strong anticoagulant effect and a high lipid solubility making the volume of distribution large. Furthermore, they have slow plasma clearance, accumulate in the liver, and undergo enterohepatic circulation. Therefore, the concentration of rat poison in the liver is high, with increased half-lives of up to 16 to 69 days. Moreover, even after the rat poison is undetectable in the serum, its anticoagulant effect can still last for days to months. ARs do not directly counteract the already synthesized clotting factors until they are relatively depleted in the body, resulting in prolonged clotting times. Therefore, bleeding symptoms tend to appear 1 to 4 weeks after taking the drug.^[14]^ In this case, the patient developed cerebral hemorrhage 5 days after indirect exposure to rat poison, and examination of coagulation parameters revealed that PT and APTT were significantly prolonged and coagulation factors VII and IX were significantly decreased, which was consistent with the report.

Currently, when vitamin K1 therapy should be discontinued in LAAR poisoning remains unclear and requires further research. Vitamin K1, as a specific antidote for AR poisoning, has a rapid onset of action against abnormal intrinsic and extrinsic coagulation pathways caused by poisoning, and coagulation function (PT, APTT, and international normalized ratio) can return to normal within 2 to 3 days after vitamin K1 treatment.^[15]^ On the other hand, second-generation ARs often accumulate in the liver and have increased half-lives of up to 16 to 220 days through elevated lipid solubility. They also have slow metabolism within specific areas. Therefore, maintenance treatment with vitamin K1 is often required after AR poisoning, and premature discontinuation of vitamin K1 may lead to abnormal coagulation and bleeding even if coagulation is normal.^[15]^ Hence, 14 days after treatment, the patient’s symptoms were significantly alleviated, with no coagulation abnormalities found on reexamination. The patient was followed up 6 months after discharge with oral administration of vitamin K1.

### 3.1. Strengths and limitations of the study

#### 3.1.1. Strengths.

Our findings suggested that rodenticide poisoning can lead to intracerebral hemorrhage and that vitamin K1 infusion is an effective treatment.

#### 3.1.2. Limitations.

The controversy regarding when to discontinue vitamin K1 therapy in LAAR poisoning remains. A study retrospectively analyzed serum rodenticide concentrations before and after oral vitamin K1 treatment in 21 patients with LAAR poisoning and found that patients with concentrations >10 μg/L were prone to rebleeding.^[16]^ For brodifacoum poisoning, it has been suggested that it is safe to discontinue vitamin K1 at serum concentrations <10 μg/L.^[17]^ In the present study, 6 months after discharge, the patient stopped taking vitamin K1 for 1 week and no coagulation abnormalities were observed. The patient then stopped taking vitamin K1 for 1 month after discharge and showed no coagulation abnormalities. Vitamin K1 was completely stopped. However, the plasma concentration of LAAR was not measured, and the risk of rebleeding exists. Future large sample-sized, multicenter, randomized controlled studies focusing on the treatment time, treatment dose, and withdrawal time should be conducted to further validate the findings of our study.

## 4. Conclusion

Although rarely reported, rodenticide poisoning can lead to cerebral hemorrhage. Moreover, because its symptoms are nonspecific, it is easy to miss the diagnosis or misdiagnose. When patients present with direct and indirect symptoms, such as dizziness, headache, and limb weakness, rodenticide poisoning should be considered. Timely examination of coagulation function and head CT or MRI examination should be performed for early diagnosis and treatment. Our findings suggested that vitamin K1 infusion is an effective treatment for rodenticide poisoning; however, the treatment time for vitamin K1 should vary from person to person.

## Author contributions

**Conceptualization:** Qian-kun He, Yuan-hua Wu.

**Data curation:** Ming-wei Liu Liu.

**Formal analysis:** Xiao-ying Lu.

**Funding acquisition:** Ming-wei Liu Liu.

**Investigation:** Yuan-hua Wu, Ming-wei Liu Liu.

**Project administration:** Xiao-ying Lu.

**Resources:** Qian-kun He, Yuan-hua Wu, Ming-wei Liu Liu.

**Software:** Qian-kun He, Xiao-ying Lu.

**Supervision:** Xiao-ying Lu, Ming-wei Liu Liu.

**Validation:** Yuan-hua Wu, Xiao-ying Lu.

**Visualization:** Qian-kun He.

**Writing – original draft:** Ming-wei Liu Liu.

**Writing – review & editing:** Yuan-hua Wu, Ming-wei Liu Liu.
